# 1,3-Dimethyl-4-phenyl­sulfanyl-1*H*-pyrazol-5-ol

**DOI:** 10.1107/S1600536811004922

**Published:** 2011-02-16

**Authors:** Tara Shahani, Hoong-Kun Fun, R. Venkat Ragavan, V. Vijayakumar, S. Sarveswari

**Affiliations:** a X-ray Crystallography Unit, School of Physics, Universiti Sains Malaysia, 11800 USM, Penang, Malaysia; b Organic Chemistry Division, School of Advanced Sciences, VIT University, Vellore 632 014, India

## Abstract

In the title compound, C_11_H_12_N_2_OS, the pyrazole ring makes a dihedral angle of 85.40 (8)° with the phenyl ring. In the crystal, inter­molecular N—H⋯O and C—H⋯O hydrogen bonds link mol­ecules into a two-dimensional network parallel to the *bc* plane.

## Related literature

For pyrazole derivatives and their microbial activity, see: Ragavan *et al.* (2009 ▶, 2010 ▶). For related structures, see: Shahani *et al.* (2009 ▶, 2010*a* ▶,*b* ▶,*c* ▶). For bond-length data, see: Allen *et al.* (1987 ▶). For the stability of the temperature controller used in the data collection, see: Cosier & Glazer (1986 ▶).
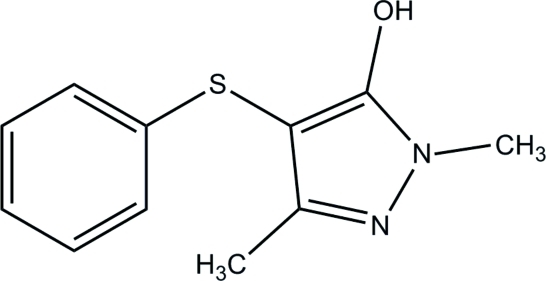


## Experimental

### 

#### Crystal data

C_11_H_12_N_2_OS
*M* *_r_* = 220.30
Orthorhombic, 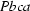

*a* = 10.9479 (2) Å
*b* = 11.3470 (3) Å
*c* = 17.7392 (4) Å
*V* = 2203.67 (9) Å^3^
*Z* = 8
Mo *K*α radiation
μ = 0.27 mm^−1^
*T* = 100 K
0.33 × 0.13 × 0.11 mm

#### Data collection

Bruker SMART APEXII CCD area-detector diffractometer
Absorption correction: multi-scan (*SADABS*; Bruker, 2009 ▶) *T*_min_ = 0.917, *T*_max_ = 0.971
12209 measured reflections
3027 independent reflections
2406 reflections with *I* > 2σ(*I*)
*R*_int_ = 0.041

#### Refinement

*R*[*F* ^2^ > 2σ(*F* ^2^)] = 0.039
*wR*(*F* ^2^) = 0.104
*S* = 1.04
3027 reflections
142 parameters
H atoms treated by a mixture of independent and constrained refinement
Δρ_max_ = 0.33 e Å^−3^
Δρ_min_ = −0.31 e Å^−3^

### 

Data collection: *APEX2* (Bruker, 2009 ▶); cell refinement: *SAINT* (Bruker, 2009 ▶); data reduction: *SAINT*; program(s) used to solve structure: *SHELXTL* (Sheldrick, 2008 ▶); program(s) used to refine structure: *SHELXTL*; molecular graphics: *SHELXTL*; software used to prepare material for publication: *SHELXTL* and *PLATON* (Spek, 2009 ▶).

## Supplementary Material

Crystal structure: contains datablocks global, I. DOI: 10.1107/S1600536811004922/is2676sup1.cif

Structure factors: contains datablocks I. DOI: 10.1107/S1600536811004922/is2676Isup2.hkl

Additional supplementary materials:  crystallographic information; 3D view; checkCIF report

## Figures and Tables

**Table 1:** Hydrogen-bond geometry (Å, °)

*D*—H⋯*A*	*D*—H	H⋯*A*	*D*⋯*A*	*D*—H⋯*A*
N1—H1*N*1⋯O1^i^	0.94 (2)	1.71 (2)	2.6446 (16)	173 (2)
C3—H3*A*⋯O1^ii^	0.93	2.53	3.2549 (19)	135

Symmetry codes: (i) 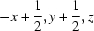; (ii) 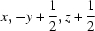.

**Fig. 1.**
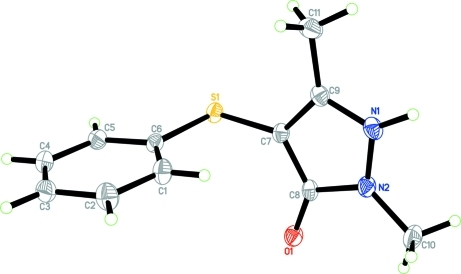
The molecular structure of the title compound, showing 50% probability displacement ellipsoids and the atom numbering scheme.

**Fig. 2.**
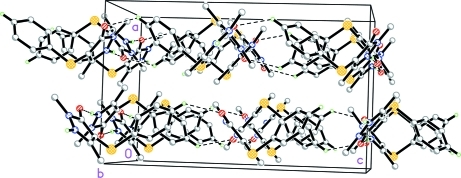
The crystal structure of the title compound viewed approximately along the b axis. Intermolecular interactions are shown in dashed lines. Hydrogen bond not involved in intermolecular interactions are omitted for clarity.

## supplementary crystallographic information

### Comment

Antibacterial and antifungal activities of the azoles are most widely studied and some of them are in clinical practice as anti-microbial agents. However, the azole-resistant strain had led to the development of new antimicrobial compounds. In particular pyrazole derivatives are extensively studied and used as antimicrobial agents. Pyrazole is an important class of heterocyclic compounds and many pyrazole derivatives are reported to have the broad spectrum of biological properties, such as anti-inflammatory, antifungal, herbicidal, anti-tumour, cytotoxic, molecular modelling, and antiviral activities. Pyrazole derivatives also act as antiangiogenic agents, A3 adenosine receptor antagonists, neuropeptide YY5 receptor antagonists, kinase inhibitor for treatment of type 2 diabetes, hyperlipidemia, obesity, and thrombopiotinmimetics. Recently urea derivatives of pyrazoles have been reported as potent inhibitors of p38 kinase. Since the high electronegativity of halogens (particularly chlorine and fluorine) in the aromatic part of the drug molecules play an important role in enhancing their biological activity, we are interested to have 4-fluoro or 4-chloro substitution in the aryls of 1,5-diaryl pyrazoles. As part of our on-going research aiming the synthesis of new antimicrobial compounds, we have reported the synthesis of novel pyrazole derivatives and their microbial activities (Ragavan *et al.*, 2009, 2010). The structure of the title compound is presented here.

In the title compound, (Fig. 1), the 1*H*-pyrazol ring (C7–C9/N1/N2) [maximum deviation of 0.00117 (14) Å] makes a dihedral angle of 85.40 (8)° with the phenyl ring (C1–C6). The bond lengths (Allen *et al.*, 1987) and angles are within normal ranges and comparable to those closely related structures (Shahani *et al.*, 2009, 2010*a*,*b*,*c*).

In the crystal packing (Fig. 2), pairs of intermolecular N1—H1N1···O1 and C3—H3A···O1 hydrogen bonds (Table 1) link the molecules into two-dimensional networks parallel to the *bc* plane.

### Experimental

The compound has been synthesized using the method available in the literature (Ragavan *et al.*, 2009) and recrystallized using the ethanol-chloroform 1:1 mixture (yield 60%, *m.p.* 444 K).

### Refinement

The H atoms bound to C atoms were positioned geometrically (C—H = 0.93–0.96 Å) with *U*_iso_(H) =1.2 or 1.5*U*_eq_(C). The H atoms attached to the N atom was located from the difference map and refined freely, [N—H = 0.94 (2) Å].

### Crystal data

**Table:**

C_11_H_12_N_2_OS	*F*(000) = 928
*M**_r_* = 220.30	*D*_x_ = 1.328 Mg m^−^^3^
Orthorhombic, *P**b**c**a*	Mo *K*α radiation, λ = 0.71073 Å
Hall symbol: -P 2ac 2ab	Cell parameters from 2873 reflections
*a* = 10.9479 (2) Å	θ = 2.8–29.1°
*b* = 11.3470 (3) Å	µ = 0.27 mm^−^^1^
*c* = 17.7392 (4) Å	*T* = 100 K
*V* = 2203.67 (9) Å^3^	Block, colourless
*Z* = 8	0.33 × 0.13 × 0.11 mm

### Data collection

**Table:**

Bruker SMART APEXII CCD area-detector diffractometer	3027 independent reflections
Radiation source: fine-focus sealed tube	2406 reflections with *I* > 2σ(*I*)
graphite	*R*_int_ = 0.041
φ and ω scans	θ_max_ = 29.4°, θ_min_ = 2.3°
Absorption correction: multi-scan (*SADABS*; Bruker, 2009)	*h* = −11→15
*T*_min_ = 0.917, *T*_max_ = 0.971	*k* = −15→15
12209 measured reflections	*l* = −16→24

### Refinement

**Table:**

Refinement on *F*^2^	Primary atom site location: structure-invariant direct methods
Least-squares matrix: full	Secondary atom site location: difference Fourier map
*R*[*F*^2^ > 2σ(*F*^2^)] = 0.039	Hydrogen site location: inferred from neighbouring sites
*wR*(*F*^2^) = 0.104	H atoms treated by a mixture of independent and constrained refinement
*S* = 1.04	*w* = 1/[σ^2^(*F*_o_^2^) + (0.0481*P*)^2^ + 0.8061*P*] where *P* = (*F*_o_^2^ + 2*F*_c_^2^)/3
3027 reflections	(Δ/σ)_max_ < 0.001
142 parameters	Δρ_max_ = 0.33 e Å^−^^3^
0 restraints	Δρ_min_ = −0.31 e Å^−^^3^

### Special details

**Table:**

Experimental. The crystal was placed in the cold stream of an Oxford Cyrosystems Cobra open-flow nitrogen cryostat (Cosier & Glazer, 1986) operating at 100.0 (1) K.
Geometry. All e.s.d.'s (except the e.s.d. in the dihedral angle between two l.s. planes) are estimated using the full covariance matrix. The cell e.s.d.'s are taken into account individually in the estimation of e.s.d.'s in distances, angles and torsion angles; correlations between e.s.d.'s in cell parameters are only used when they are defined by crystal symmetry. An approximate (isotropic) treatment of cell e.s.d.'s is used for estimating e.s.d.'s involving l.s. planes.
Refinement. Refinement of *F*^2^ against ALL reflections. The weighted *R*-factor *wR* and goodness of fit *S* are based on *F*^2^, conventional *R*-factors *R* are based on *F*, with *F* set to zero for negative *F*^2^. The threshold expression of *F*^2^ > σ(*F*^2^) is used only for calculating *R*-factors(gt) *etc*. and is not relevant to the choice of reflections for refinement. *R*-factors based on *F*^2^ are statistically about twice as large as those based on *F*, and *R*- factors based on ALL data will be even larger.

### Fractional atomic coordinates and isotropic or equivalent isotropic displacement parameters (Å^2^)

**Table:**

	*x*	*y*	*z*	*U*_iso_*/*U*_eq_	
S1	0.09449 (3)	0.31932 (3)	0.09298 (2)	0.01802 (11)	
O1	0.32686 (10)	0.34450 (8)	−0.02532 (6)	0.0207 (2)	
N1	0.20240 (12)	0.61926 (10)	0.00845 (8)	0.0202 (3)	
N2	0.28173 (12)	0.54374 (10)	−0.02791 (7)	0.0192 (3)	
C1	0.28747 (15)	0.36136 (13)	0.19278 (9)	0.0225 (3)	
H1A	0.3057	0.4285	0.1649	0.027*	
C2	0.35663 (15)	0.33304 (13)	0.25590 (9)	0.0254 (3)	
H2A	0.4211	0.3816	0.2700	0.031*	
C3	0.33066 (15)	0.23327 (14)	0.29808 (9)	0.0236 (3)	
H3A	0.3766	0.2154	0.3407	0.028*	
C4	0.23531 (15)	0.16043 (13)	0.27606 (9)	0.0243 (3)	
H4A	0.2180	0.0929	0.3037	0.029*	
C5	0.16551 (14)	0.18758 (13)	0.21304 (9)	0.0212 (3)	
H5A	0.1019	0.1382	0.1986	0.025*	
C6	0.19092 (13)	0.28904 (12)	0.17148 (8)	0.0173 (3)	
C7	0.16428 (13)	0.43840 (11)	0.04922 (8)	0.0166 (3)	
C8	0.26321 (13)	0.43076 (11)	−0.00304 (8)	0.0164 (3)	
C9	0.13144 (14)	0.55679 (12)	0.05474 (8)	0.0178 (3)	
C10	0.38173 (15)	0.58669 (13)	−0.07387 (10)	0.0239 (3)	
H10A	0.4171	0.5222	−0.1013	0.036*	
H10B	0.4427	0.6217	−0.0420	0.036*	
H10C	0.3517	0.6446	−0.1087	0.036*	
C11	0.03683 (15)	0.61354 (14)	0.10271 (9)	0.0248 (3)	
H11A	0.0198	0.6912	0.0839	0.037*	
H11B	0.0661	0.6189	0.1536	0.037*	
H11C	−0.0365	0.5672	0.1016	0.037*	
H1N1	0.1983 (19)	0.700 (2)	−0.0036 (13)	0.047 (6)*	

### Atomic displacement parameters (Å^2^)

**Table:**

	*U*^11^	*U*^22^	*U*^33^	*U*^12^	*U*^13^	*U*^23^
S1	0.01876 (19)	0.01847 (18)	0.0168 (2)	−0.00372 (13)	−0.00109 (13)	0.00267 (13)
O1	0.0267 (6)	0.0139 (4)	0.0215 (6)	0.0008 (4)	0.0046 (4)	0.0000 (4)
N1	0.0264 (7)	0.0129 (5)	0.0212 (7)	0.0017 (5)	−0.0005 (5)	−0.0006 (5)
N2	0.0240 (6)	0.0131 (5)	0.0206 (7)	−0.0002 (5)	0.0034 (5)	0.0007 (5)
C1	0.0273 (8)	0.0198 (7)	0.0204 (8)	−0.0046 (6)	−0.0027 (6)	0.0034 (6)
C2	0.0285 (8)	0.0257 (7)	0.0221 (9)	−0.0037 (6)	−0.0065 (7)	−0.0002 (6)
C3	0.0255 (8)	0.0298 (8)	0.0155 (8)	0.0053 (6)	−0.0012 (6)	0.0006 (6)
C4	0.0247 (8)	0.0260 (7)	0.0222 (8)	0.0015 (6)	0.0032 (6)	0.0082 (6)
C5	0.0197 (7)	0.0213 (7)	0.0225 (8)	−0.0015 (6)	0.0013 (6)	0.0043 (6)
C6	0.0196 (7)	0.0188 (6)	0.0134 (7)	0.0007 (5)	0.0019 (5)	0.0003 (5)
C7	0.0193 (7)	0.0148 (6)	0.0156 (7)	−0.0014 (5)	−0.0003 (5)	0.0005 (5)
C8	0.0224 (7)	0.0127 (6)	0.0141 (7)	−0.0017 (5)	−0.0018 (6)	0.0001 (5)
C9	0.0207 (7)	0.0179 (6)	0.0149 (7)	0.0009 (5)	−0.0035 (6)	−0.0002 (5)
C10	0.0283 (8)	0.0184 (7)	0.0250 (8)	−0.0043 (6)	0.0050 (7)	0.0032 (6)
C11	0.0248 (8)	0.0250 (7)	0.0245 (9)	0.0071 (6)	−0.0008 (6)	−0.0021 (6)

### Geometric parameters (Å, °)

**Table:**

S1—C7	1.7356 (14)	C3—H3A	0.9300
S1—C6	1.7809 (15)	C4—C5	1.389 (2)
O1—C8	1.2648 (17)	C4—H4A	0.9300
N1—C9	1.3343 (19)	C5—C6	1.395 (2)
N1—N2	1.3801 (17)	C5—H5A	0.9300
N1—H1N1	0.94 (2)	C7—C9	1.3942 (19)
N2—C8	1.3708 (17)	C7—C8	1.428 (2)
N2—C10	1.4494 (19)	C9—C11	1.487 (2)
C1—C2	1.389 (2)	C10—H10A	0.9600
C1—C6	1.391 (2)	C10—H10B	0.9600
C1—H1A	0.9300	C10—H10C	0.9600
C2—C3	1.387 (2)	C11—H11A	0.9600
C2—H2A	0.9300	C11—H11B	0.9600
C3—C4	1.388 (2)	C11—H11C	0.9600
			
C7—S1—C6	103.83 (7)	C1—C6—S1	123.32 (11)
C9—N1—N2	108.92 (11)	C5—C6—S1	117.02 (11)
C9—N1—H1N1	129.0 (13)	C9—C7—C8	107.44 (12)
N2—N1—H1N1	121.9 (13)	C9—C7—S1	127.24 (12)
C8—N2—N1	109.70 (12)	C8—C7—S1	125.24 (10)
C8—N2—C10	127.39 (13)	O1—C8—N2	122.79 (13)
N1—N2—C10	121.97 (11)	O1—C8—C7	131.88 (13)
C2—C1—C6	119.79 (14)	N2—C8—C7	105.33 (12)
C2—C1—H1A	120.1	N1—C9—C7	108.57 (13)
C6—C1—H1A	120.1	N1—C9—C11	121.85 (13)
C3—C2—C1	120.80 (15)	C7—C9—C11	129.57 (14)
C3—C2—H2A	119.6	N2—C10—H10A	109.5
C1—C2—H2A	119.6	N2—C10—H10B	109.5
C2—C3—C4	119.26 (15)	H10A—C10—H10B	109.5
C2—C3—H3A	120.4	N2—C10—H10C	109.5
C4—C3—H3A	120.4	H10A—C10—H10C	109.5
C3—C4—C5	120.56 (14)	H10B—C10—H10C	109.5
C3—C4—H4A	119.7	C9—C11—H11A	109.5
C5—C4—H4A	119.7	C9—C11—H11B	109.5
C4—C5—C6	119.92 (14)	H11A—C11—H11B	109.5
C4—C5—H5A	120.0	C9—C11—H11C	109.5
C6—C5—H5A	120.0	H11A—C11—H11C	109.5
C1—C6—C5	119.66 (14)	H11B—C11—H11C	109.5
			
C9—N1—N2—C8	−1.48 (17)	N1—N2—C8—O1	−177.46 (13)
C9—N1—N2—C10	−171.15 (14)	C10—N2—C8—O1	−8.5 (2)
C6—C1—C2—C3	0.0 (2)	N1—N2—C8—C7	2.10 (16)
C1—C2—C3—C4	0.9 (2)	C10—N2—C8—C7	171.06 (14)
C2—C3—C4—C5	−0.8 (2)	C9—C7—C8—O1	177.55 (16)
C3—C4—C5—C6	−0.2 (2)	S1—C7—C8—O1	−5.6 (2)
C2—C1—C6—C5	−1.0 (2)	C9—C7—C8—N2	−1.96 (16)
C2—C1—C6—S1	178.43 (12)	S1—C7—C8—N2	174.91 (11)
C4—C5—C6—C1	1.1 (2)	N2—N1—C9—C7	0.18 (17)
C4—C5—C6—S1	−178.38 (12)	N2—N1—C9—C11	179.36 (13)
C7—S1—C6—C1	7.87 (15)	C8—C7—C9—N1	1.12 (17)
C7—S1—C6—C5	−172.70 (12)	S1—C7—C9—N1	−175.67 (11)
C6—S1—C7—C9	−100.38 (14)	C8—C7—C9—C11	−177.97 (15)
C6—S1—C7—C8	83.36 (14)	S1—C7—C9—C11	5.2 (2)

### Hydrogen-bond geometry (Å, °)

**Table:**

*D*—H···*A*	*D*—H	H···*A*	*D*···*A*	*D*—H···*A*
N1—H1N1···O1^i^	0.94 (2)	1.71 (2)	2.6446 (16)	173 (2)
C3—H3A···O1^ii^	0.93	2.53	3.2549 (19)	135

Symmetry codes: (i) −*x*+1/2, *y*+1/2, *z*; (ii) *x*, −*y*+1/2, *z*+1/2.
